# Enhancing students’ engagement on using Blackboard as an online learning community tool in Clinical Biochemistry

**DOI:** 10.15694/mep.2020.000071.1

**Published:** 2020-04-20

**Authors:** Ayman Elsamanoudy, Fayza Al Fayez, Aliaa Alamoudi, Mohammed Hassanien

**Affiliations:** 1 Medical Biochemistry and Molecular Biology; 2 Clinical Biochemistry; 3 Clinical Biochemistry; 4 Vice Presidency for educational Affairs and College of Pharmacy

**Keywords:** Blackboard, online learning community, engagement, perception

## Abstract

This article was migrated. The article was marked as recommended.

**Background and aim of the study:** Proper online students’/teacher communication and awareness of the use of Blackboard as an online learning community could result in the improvement of student academic achievement. So, the aim of the current project was directed to encourage students and faculty for using Blackboard as an online learning community tool. Moreover, it aimed to measures their perceptions.

**Subjects and Methods:** The study was directed to the second year Medical students during teaching of Clinical Biochemistry. We started by pre-implementation survey, followed by orientation workshop, engaging students for Blackboard implementation then ended in post-implementation survey to evaluate how come students are engaged and satisfied.

**Results:** The first questionnaire group indicated the students’ perceptions of Blackboard use and usefulness as an online learning community tool. The second questionnaire group was related to the Interest & Enjoyment of using Backboard. While Blackboard Tools are presented in the third questionnaire questions group. The comparison regarding the total perception of the three groups of the questionnaire between pre-implementation versus post-implementation survey results showed significant improvement of students’ perception in the post-implementation phase. By correlating grades in Clinical Biochemistry and total GPA to the questionnaire groups, total perceptions showed a non-significant correlation between all studied parameters in the pre-implementation phase while significant positive correlations were detected in the post-implementation phase.

**Conclusion:** Based on the presented second-year medical students’ perception and satisfaction, we could confirm that the Blackboard online platform is a very successful online line learning community tool. Blackboard together with face to face classroom-based learning methods helps students for better learning achievement. Moreover, the online learning community is considered a complementary tool to that of classroom-based learning as it facilitates students/students and students’/faculty communication and minimizes the time required for physical contacts.

## Introduction

### Introduction and Aim of the work

Professional learning communities(PLCs) refer to the concept of the common goal of “community” models. It aims to promote collaboration among teachers and students by creating a collaborative professional culture ( Stoll *et al.*, 2006). It offers an infrastructure to create the supportive cultures and conditions necessary for achieving significant gains in teaching and learning. Moreover, PLCs assist teachers to become more effective in their work and communication with their students ( Morrissey, 2000).These learning communities may consist of teachers who share subjects, students, or grade levels ( Vangrieken *et al.*, 2015).

The use of e-learning has been dominated by many educational organizations. E-learning is used even with full distance learning or as a supplement with classroom education. A wide set of Learning Management Systems (LMSs) have been developed and used to support the e-learning process. LMSs- which are mainly internet based- being software allowing instructors to manage materials distribution, assignments, communications and other aspects of instructions for their courses ( Epstein *et al.*, 2013).

Today, LMSs have become an integral component of the educational systems in most universities ( Pishva, Nishantha and Dang, 2010). It involves Hybrid courses that consist of both web-based and classroom sessions, with a varying degree of time allotted to the online and in-class sessions, depending upon the nature of the class and discretion of the instructor ( Pishva, Nishantha and Dang, 2010).

An example of the most widely used online learning community tool which usually used in a formal way is Blackboard. Another widely used but usually non-formal is the use of social media. Blackboard is one of the most common web-based LMS that is developed and maintained by Blackboard Inc. It is an entirely web-based learning platform. It is used for communication between teachers and students as well as providing a storage place for all types of information. Blackboard also contains a number of administrative tools to support the student and teacher in their work ( Spelke, 2011).

Since the poor online students /teacher communication with the insufficient contact time, good awareness of the use of Blackboard as an online learning community in the core course of Clinical Biochemistry-Faculty of Medicine/King Abdulaziz University(FOM/KAU) could result in improvement of student satisfaction and academic achievement. Moreover, it will affect curricular changes and faculty as well as program development.

So, the aim of the current project is directed to encourage students and faculty for using a blackboard as an online learning community tool. To achieve this aim, the research seeks to measures students’ perception at the start of the project, implementing orientation workshops directed to both students and faculty, then, re-evaluating students’ perception in a post-implementation stage. The main mission of the study deals with the role of Blackboard as an online learning community context tool beside face-to-face education inside the traditional classroom.

## Methods

This study was carried out in the faculty of Medicine, King Abdulaziz University, Saudi Arabia. It is approved by the Biomedical Ethics Research committee of King Abdulaziz University (Reference No 185-19).

The study was directed to the students of second year Medicine during teaching of Clinical Biochemistry course. We started by pre-implementation survey to measure the students’ perception as a base line for our study (167 males and 71 females) followed by orientation workshop, engaging students for Blackboard implementation then ended in post-implementation survey to evaluate how come students are engaged and satisfied (137 males and 63 females). A number of students are not included in the second survey (40 males and 8 females).

Blackboard is the online learning platform that is documented for students and supported by E- learning unit. Blackboard has been developed and maintained by Blackboard Inc., is one of the most common web-based LMS ( Spelke, 2011).

There are many tools available on Blackboard for both teachers and students. These include; Home page, Course & Learning Materials, Announcements, Discussion Boards, Messages, Roster, My Groups, My grades (Grade center) and The assessment tools that include interactive formative exams, assignments, Quizzes, and tests. Moreover, many students supporting tools are also available as Contact professors, Students supports and Professor resources. The virtual classroom is also one of the Blackboard teaching tools.

The research project was classified into Four stages:

1. Pre-implementation survey.
2. Pre-implementation workshops (Two workshops; the first is directed to the faculty and the second is directed to students).
3. Engagement phase.
4. Post-implementation survey.

### Orientation Workshop

The workshops were conducted as a part of professional development training program in collaboration with e learning unit in King Abulaziz University-Faculty of Medicine. Workshops were carried out as face to face training for staff (one to one) and to students as team work in E-learning unit in multiple sessions (10 students /session).

Staff workshops aimed to gain the following outcomes:

1. Create content items to deliver many learning activities, to engage students and enhance their learning process.
2. Construct different forms of assessments methods (assignment and formative exams).
3. Record data, grades, and monitor student achievement and progress.
4. Effectively utilize collaboration tools and online communication to enhance student engagement by providing different means to keep in contact with students (Discussion Board, Forum and Students’ Support).

Students workshops aimed to gain the following outcomes:

1. Inform students about the importance of the online learning community in teaching and learning.
2. Provide students with full detailed methods and tools of Blackboard as one of the online learning community tools.
3. Effectively utilize Blackboard utilities in a perfect manner especially those that provide good communication with their staff.

### Surveys

The study survey was constructed and validated to test students’ perceptions of Blackboard use. It is applied twice (pre-implementation and post-implementation).

Survey was designed according to that published previously by ( Carvalho, Areal and Silva, 2011). It is classified into three questionnaire groups: First questionnaire group for Blackboard usage & usefulness (10 questions), second questionnaire group for interest & enjoyment (5 questions) and third questionnaire group for Blackboard tools (6 questions).

Five-point Likert scale was used with 1 has the lowest scale and 5 has the greatest one [1; strongly disagree, 2; disagree, 3; neutral, 4; agree, 5; strongly agree].

Students were initially proved that they had a good understanding of the background, scenario, and issue regarding its content. Online google doc for questionnaire distribution was used at the following link:

https://docs.google.com/forms/d/e/1FAIpQLSfBetaKH6ONaSvDvut9xxQOM1UfY1mBW4egTT_grx-65AOU9A/viewform?usp=sf_link

### Statistical methods

Data were collected as google documents and excel sheets. Data were analyzed using Statistical Package for Social Science software computer program version 23 (SPSS, Inc., Chicago, IL, USA). Quantitative parametric data were presented in mean and standard deviation, while Quantitative non-parametric data were presented in median & range. Qualitative data were presented as frequency (Number-percent). Student’s t-test was used for comparison between groups with parametric data (unpaired test; for two different groups and paired tests; for related groups). One-way ANOVA followed by post-hoc Tukey was used to compare more than two different groups with parametric data. While a Mann-Whitney test was used for those with non-parametric data. Kruskal Wallis followed by post-Hoc Dunn’s was used to compare between more than two different groups with non-parametric data. Pearson’s correlation test was used to correlate between totals of questionnaires while spearman’s correlation was used to correlate between totals of questionnaires & GPA and grade. Cronbach’s Alpha test was used to assess the reliability of questionnaires. A P-value of less than 0.05 was considered statistically significant.

## Results/Analysis

The study is conducted to students of second-year Medicine during the teaching of Clinical Biochemistry. The number of students, their grades in Clinical Biochemistry course and the total GPA are presented in table (1). The number of students participating in the post-implementation (137 males and 63 females) is much lower than those of pre-implementation survey (167 males and 71 females) with missing of 40 males and 8 females as they didn’t complete the study.

**Table 1.** Gender, Grades in clinical biochemistry and Total GPA of the participants

		Pre-Implementation		Post- Implementation	
		No	%	No	%
**Gender**	**Male**	**167**	**70.2%**	**137**	**68.5%**
	**Female**	**71**	**29.8%**	**63**	**31.5%**
**Grade in Clinical Biochemistry**	**A**	**36**	**15.1%**	**35**	**17.5%**
	**A+**	**27**	**11.3%**	**21**	**10.5%**
	**B**	**44**	**18.5%**	**40**	**20%**
	**B+**	**44**	**18.5%**	**39**	**19.5%**
	**C**	**17**	**7.1%**	**12**	**6%**
	**C+**	**31**	**13.0%**	**32**	**16%**
	**D**	**15**	**6.3%**	**11**	**5.5%**
	**D+**	**24**	**10.1%**	**10**	**5%**
**Total GPA**	**2.5-3.49**	**11**	**4.6%**	**8**	**4%**
	**3.5-4.49**	**103**	**43.3%**	**107**	**53.5%**
	**>4.5**	**124**	**52.1%**	**85**	**42.5%**

Data are presented as number(No) and Percentage(%).

The results of the reliability testing are presented in table (2) as Cronbach’s Alpha test was used to assess the reliability of questionnaires. All components of the questionnaire sectors give good reliability as indicated in the table (for pre and post implementation survey groups).

**Table 2.** Reliability tests of the perception questionnaires (pre-implementation and post-implementation students’ perception)

	N of Items	Cronbach’s Alpha	Mean	Variance	Std. Deviation
Pre-implementation students ‘perception					
**First**	**10**	**0.935**	**20.277**	**105.551**	**10.2738**
**Second**	**5**	**0.899**	**10.807**	**29.954**	**5.4730**
**Third**	**6**	**0.839**	**11.702**	**32.632**	**5.7125**
Post-implementation students ‘perception					
**First**	**10**	**0.84**	**27.355**	**62.823**	**7.9261**
**Second**	**5**	**0.82**	**14.625**	**15.160**	**3.8936**
**Third**	**6**	**0.79**	**20.375**	**12.517**	**3.5379**

Cronbach’s Alpha test was used to assess reliability of questionnaires

Data of questionnaire questions are presented as median and range in tables ( 3, 4 & 5). Table (3) shows items (1-12) regardingthe results related to the first questionnaire group (Usage and usefulness). It indicates the students ‘perceptions of Blackboard use and usefulness as an online learning community tool. The second questionnaire group is related to the Interest & Enjoyment of using Backboard (items from 1-6) ( table 4). While Blackboard Tools are presented in the third questionnaire questions group (items from 1-5) ( table 5).

**Table 3.** Pre-implementation and post-implementation students’ perception for Blackboard usage and usefulness

		Pre-Implementation		Post- Implementation	
		Median	Range	Median	Range
	**First Questionnaire[ Usage & Usefulness]**				
**Q1**	I have used Blackboard to stay in contact with the Biochemistry course	**1.0**	**1.0**- **5.0**	**3.0**	**1.0-5.0**
**Q2**	I have used the learning resources available on Blackboard to develop my subject understanding	**1.0**	**1.0**- **5.0**	**3.0**	**1.0-5.0**
**Q3**	Using Blackboard has helped to develop more effective study methods	**1.0**	**1.0**- **5.0**	**3.0**	**1.0-5.0**
**Q4**	Blackboard is useful as a 24/7 ‘one stop shop’ to catch up on missed work and lectures	**2.0**	**1.0**- **5.0**	**2.0**	**1.0-5.0**
**Q5**	Blackboard has enabled me to understand what is expected of me to succeed in the course	**2.0**	**1.0**- **5.0**	**2.0**	**1.0-5.0**
**Q6**	I have used Blackboard to test my understanding of the meaning of concepts	**1.0**	**1.0**- **5.0**	**2.0**	**1.0-5.0**
**Q7**	Blackboard has been useful in allowing me to understand the assessment requirements of a module	**2.0**	**1.0**- **5.0**	**4.0**	**1.0-5.0**
**Q8**	Blackboard has allowed me to learn at my own pace	**1.0**	**1.0**- **5.0**	**2.0**	**1.0-5.0**
**Q9**	Blackboard has enabled me to make decisions about what to learn and when	**1.0**	**1.0**- **5.0**	**3.0**	**1.0-5.0**
**Q10**	Blackboard is most useful as a revision aid.	**1.0**	**1.0**- **5.0**	**5.0**	**1.0-5.0**

Data are presented as Median and Range [nonparametric data].

Kruskal Wallis followed by post-hoc Dunn’s was used to compare between more than two different groups [non-parametric data].

**Table 4.** Pre-implementation and post-implementation students’ perception for Blackboard Interest and Enjoyment

		Pre-Implementation		Post- Implementation	
		Median	Range	Median	Range
	**Second Questionnaire [Interest & Enjoyment]**				
**Q1**	Blackboard tells me all I need to know on a given topic	**2.0**	**1.0-5.0**	**2.0**	**1.0-5.0**
**Q2**	I have enjoyed working with Blackboard	**2.0**	**1.0-5.0**	**2.0**	**1.0-5.0**
**Q3**	Using Blackboard has enabled me to develop my interest in a subject	**1.0**	**1.0-5.0**	**3.0**	**1.0-5.0**
**Q4**	I would like to do other modules and core courses that use Blackboard	**2.0**	**1.0-5.0**	**5.0**	**1.0-5.0**
**Q5**	Overall I believe that Blackboard has made a positive contribution to my learning	**2.0**	**1.0-5.0**	**4.0**	**1.0-5.0**

Data are presented as Median and Range [nonparametric data].

Kruskal Wallis followed by post-hoc Dunn’s was used to compare between more than two different groups [non-parametric data].

**Table 5.** Pre-implementation and post-implementation students’ perception for Blackboard tools

		Pre-Implementation		Post- Implementation	
		Median	Range	Median	Range
	**Third Questionnaire [Blackboard Tools]**				
**Q1**	I used discussion board tool in blackboard	**1.0**	**1.0-5.0**	**4.0**	**1.0-5.0**
**Q2**	I used the forum tool in blackboard	**1.0**	**1.0-5.0**	**4.0**	**1.0-5.0**
**Q3**	I used the degree center in blackboard to track my achievement	**2.0**	**1.0-5.0**	**5.0**	**1.0-5.0**
**Q4**	I used the virtual classroom tool in blackboard	**1.0**	**1.0-5.0**	**4.0**	**1.0-5.0**
**Q5**	I know the benefits from students support and professor resources tool in blackboard	**1.0**	**1.0-5.0**	**3.0**	**1.0-5.0**
**Q6**	Overall I believe that Blackboard has made a positive contribution to my learning	**2.0**	**1.0-5.0**	**3.0**	**1.0-5.0**

Data are presented as Median and Range [nonparametric data].

Kruskal Wallis followed by post-hoc Dunn’s was used to compare between more than two different groups [non-parametric data].

**Figure 1.**
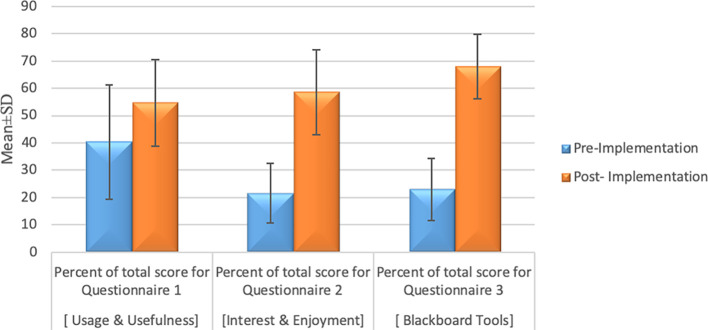
Comparison of pre-implementation and post-implementation students’ perception. ANAOVA followed by post-hoc Tukey was used to compare more than two different groups [parametric data].

The comparison regarding the total perception of the three groups of the questionnaire between pre-implementation versus post-implementation survey results are presented in Figure (1). It shows significant improvement of students ‘perception in the post-implementation phase (after orientation workshop and students engagements of using Blackboard) in comparison to that of pre- implementation phase.

Correlating grades in Clinical Biochemistry and total GPA to the questionnaire groups total perceptions are presented in Figure (2). It shows a non-significant correlation between all studied parameters in the pre-implementation phase while significant positive correlations are detected in the post-implementation phase.

**Figure 2a.**
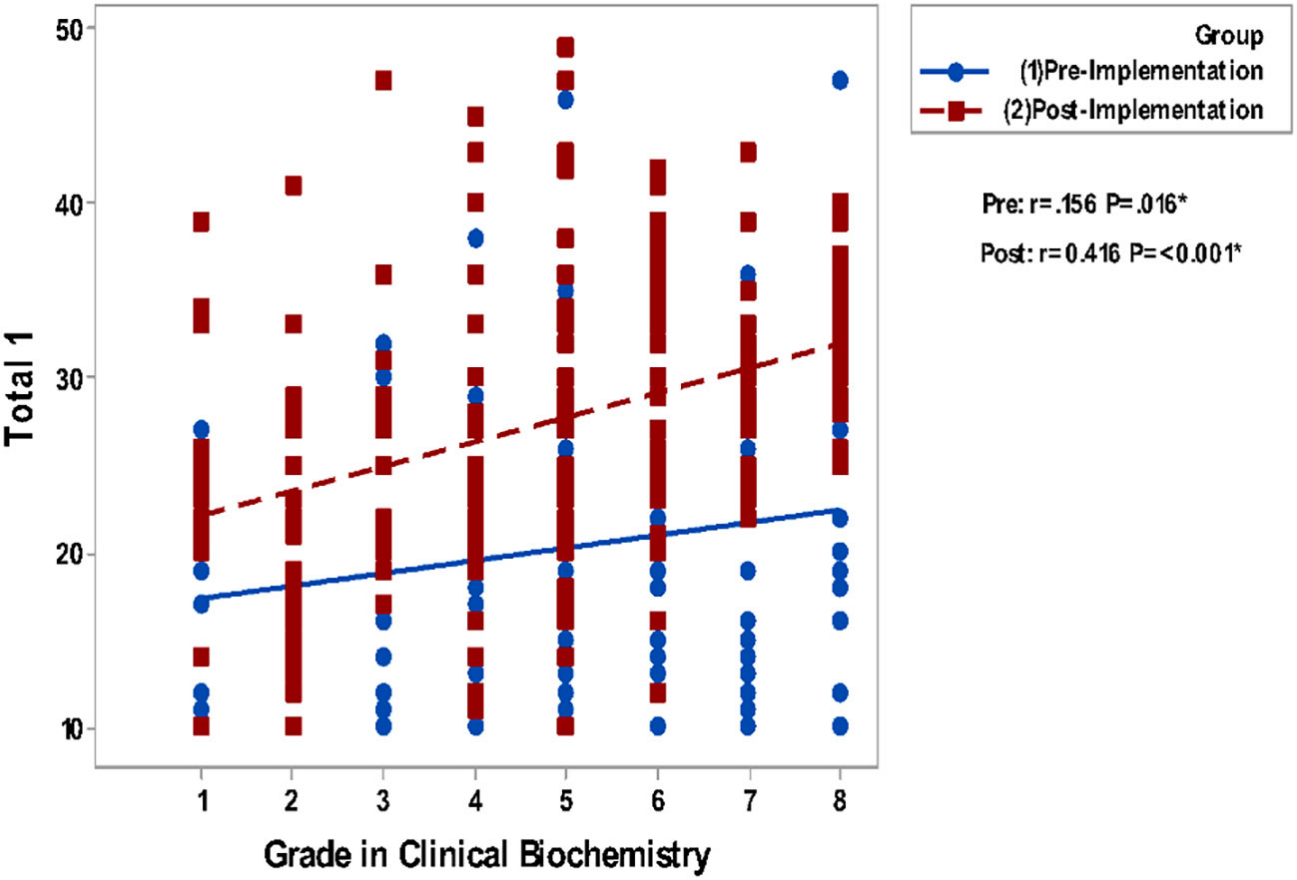
Correlation of Blackboard Usage & Usefulness (total 1) with Grades in Biochemistry. Spearman’s correlation was used to correlate between totals of questionnaires & gender, GPA and grade.

**Figure 2b.**
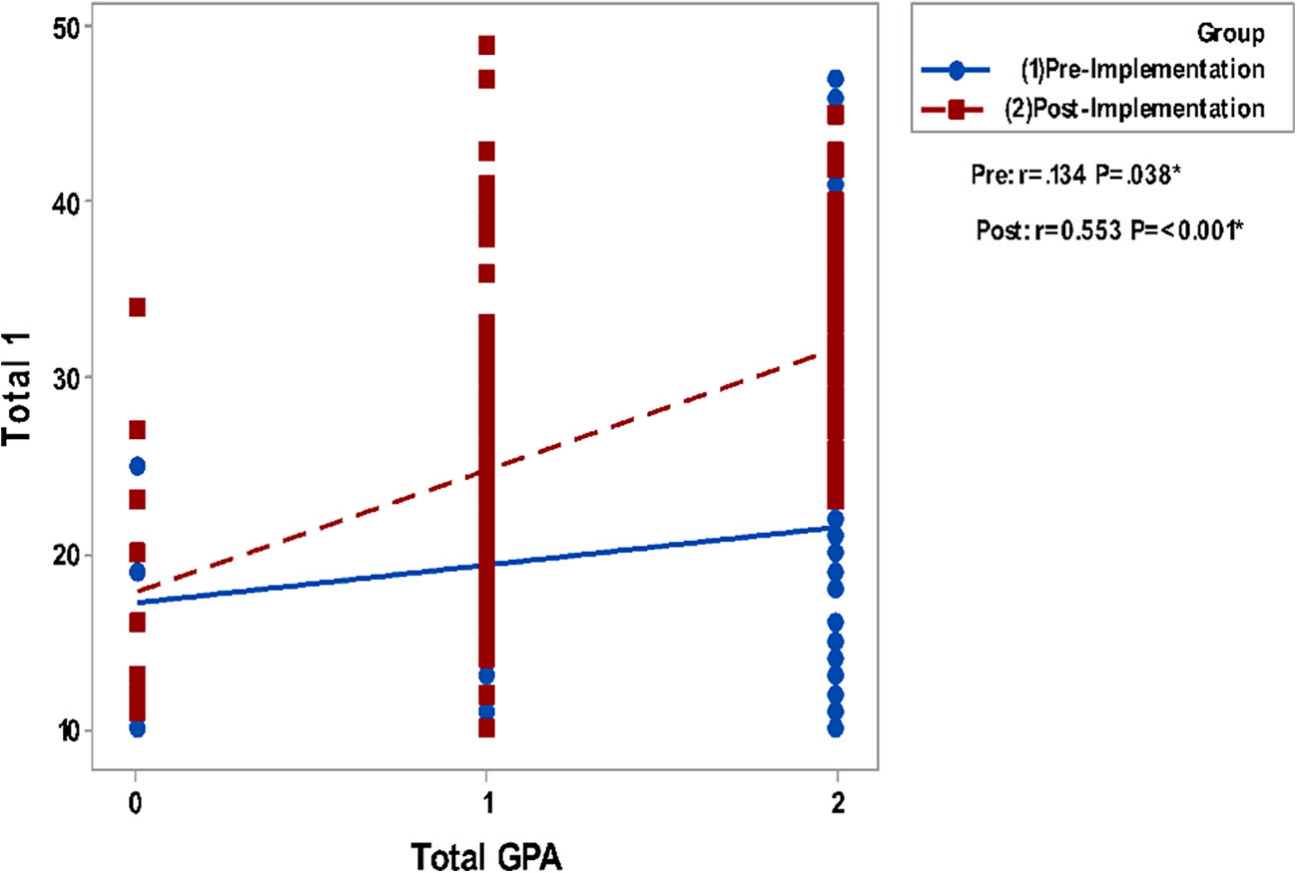
Correlation of Blackboard Usage & Usefulness (total 1) with Total GPA. Spearman’s correlation was used to correlate between totals of questionnaires & gender, GPA and grade.

**Figure 2c.**
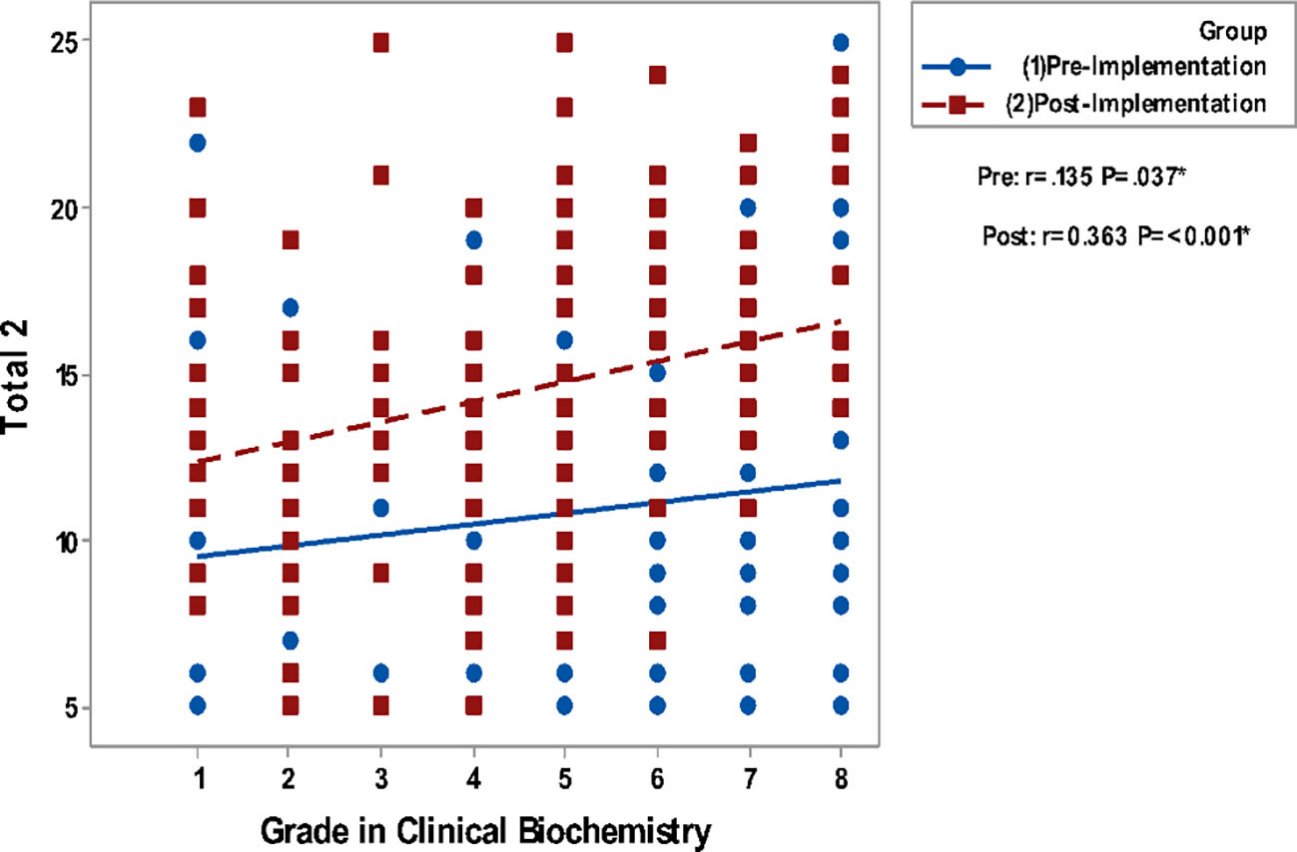
Correlation of Blackboard Interest & Enjoyment (total 2) with Grades in Biochemistry. Spearman’s correlation was used to correlate between totals of questionnaires & gender, GPA and grade.

**Figure 2d.**
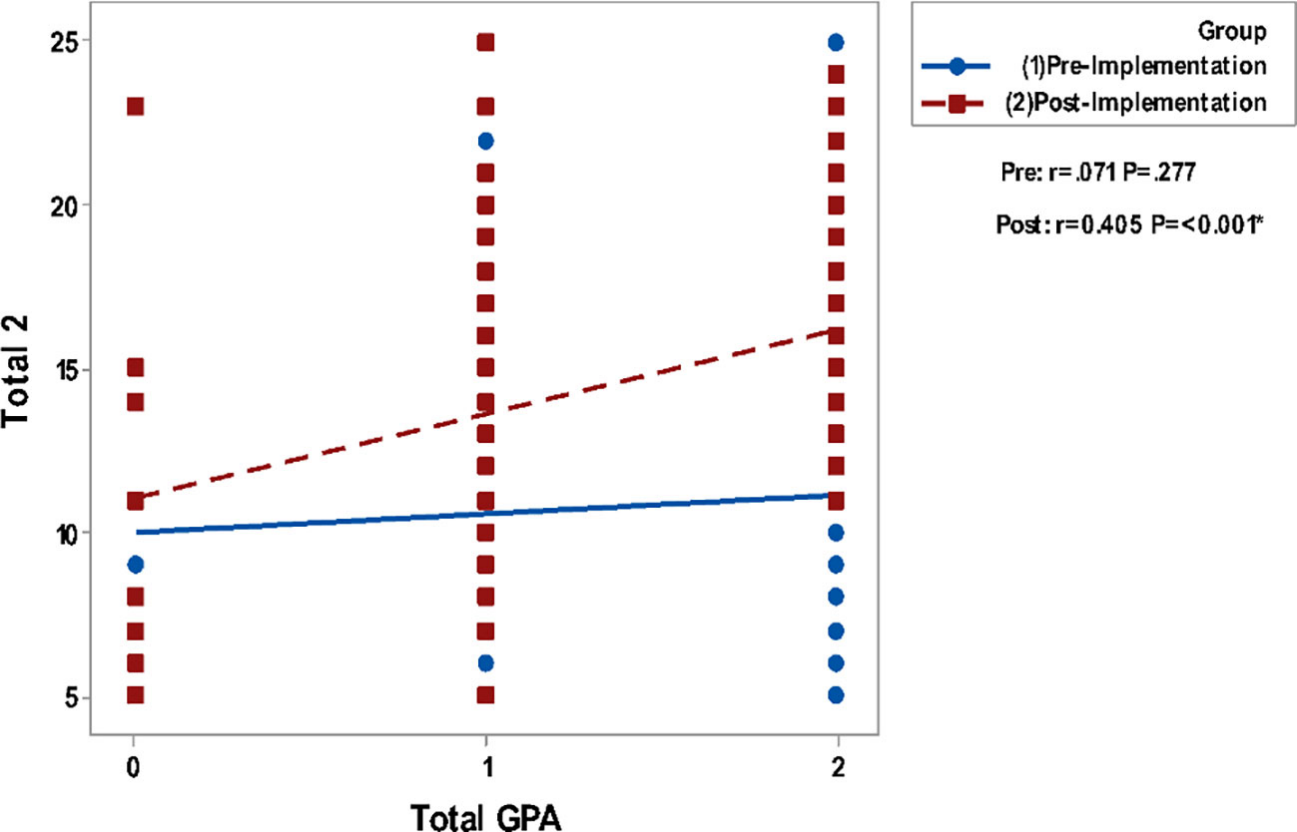
Correlation of Blackboard Interest & Enjoyment (total 2) with Total GPA. Spearman’s correlation was used to correlate between totals of questionnaires & gender, GPA and grade.

**Figure 2e.**
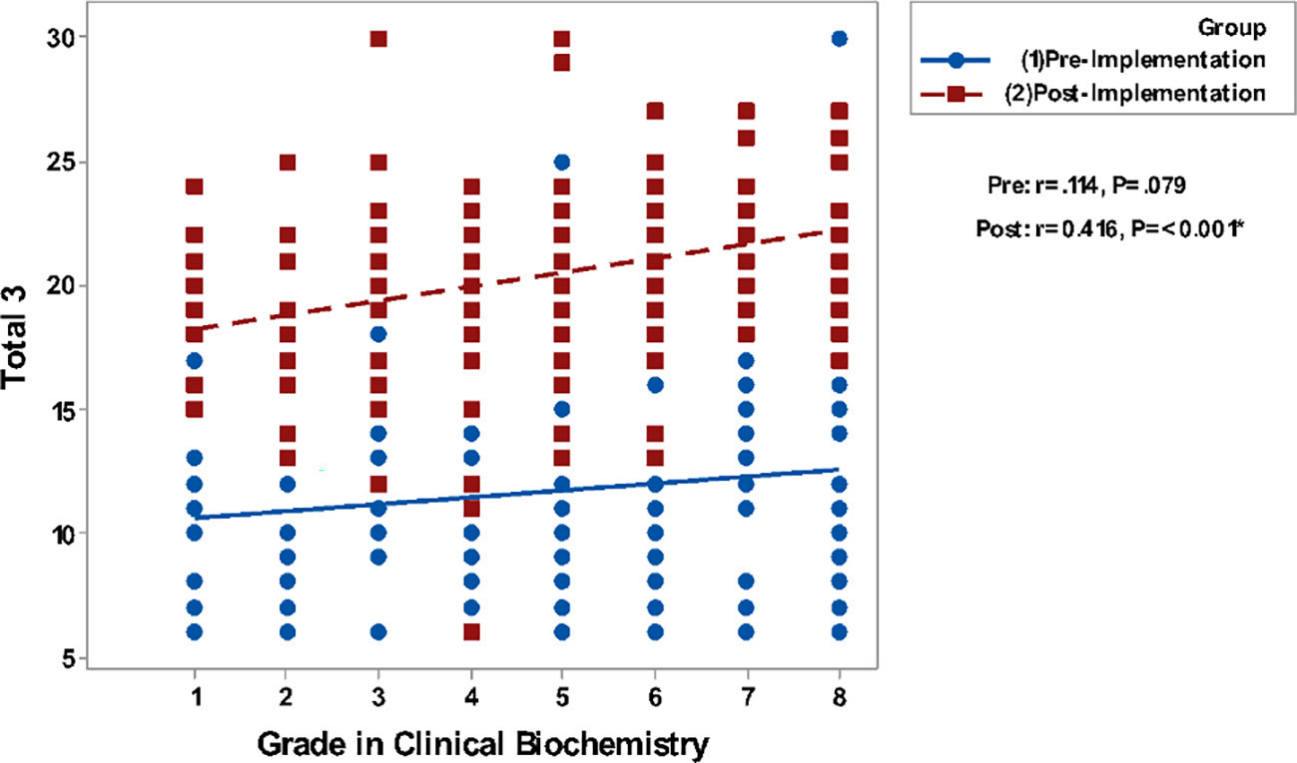
Correlation of Blackboard tools (total 3) with Grades in Biochemistry. Spearman’s correlation was used to correlate between totals of questionnaires & gender, GPA and grade.

**Figure 2f.**
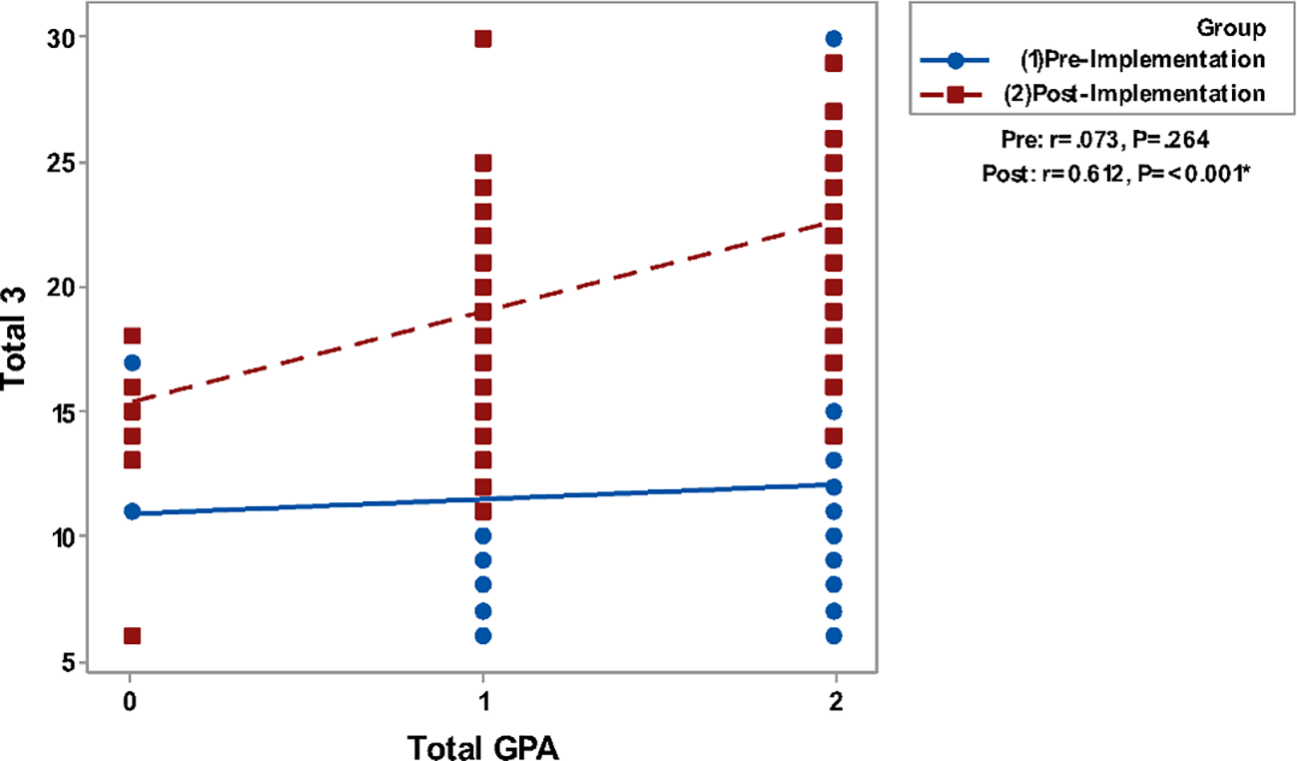
Correlation of Blackboard tools (total 3) with Total GPA. Spearman’s correlation was used to correlate between totals of questionnaires & gender, GPA and grade.

## Discussion

The use of e-learning has been dominated in many educational organizations. E-learning is used even with full distance learning or as a supplement with class room education. A wide set of Learning Management Systems (LMSs) have been developed and used to support the e-leaning process. From educational point of view, e-learning platforms are also known as Learning Management Systems (LMSs) which are “internet based, software allowing instructors to manage materials distribution, assignments, communications and other aspects of instructions for their courses” ( Epstein *et al.*, 2013). A LMS is not intended to replace the traditional classroom setting, but its main role is to supplement the traditional lecture with course content that can be accessed from campus or the Internet ( Landry, Griffeth and Hartman, 2006).

Based on this previously published literature we aimed to enhance students and faculty for using a blackboard as an online learning community tool. Moreover, it aims to measure the degree of perception and satisfaction of students after implementing an awareness program to help their engagement in using Blackboard as an online learning tool.

Measuring students’ perception and satisfaction was performed twice; at the start of the study, then repeated again after implementing the awareness workshop for both students and faculty and students’ engagement. A comparison between the two surveys was done. The survey that was used in the current study is composed of three questionnaire groups; the first questionnaire group of questions assesses the students’ awareness and perception about the Blackboard usage and usefulness, the second questionnaire group evaluates how is the use of Blackboard interested while, the third questionnaire group evaluates students’ awareness and utilization of the different learning, assessment and communication tools of Blackboard.

One of the identified constraints we faced is the difficulty and unfamiliarity of using online learning management system by the faculty as reported by ( Bradford *et al.*, 2007). The orientation workshop for faculty was mandatory to overcome this problem in our study. Moreover, staff members need to understand the pedagogical basis of the use of learning management systems and the significance of its implementation regarding students’ performance and the ease of communication with them ( Christie and Jurado, 2009). Teacher also, should know and understand adult learning requirements and the concept of Andragogy ( Bierema, 2014) & ( Reinders *et al.*, 2014). Orientation workshops dealt with the concept of that E-learning differs fundamentally from the traditional way of teaching, so it requires major commitment of time and training.

Regarding students, the positive and significant relationship between students and their teachers clarify the importance of learner-instructor, and learner-learner interactions. That can be provided by Web-based learning tool ( Sher, 2009) like Blackboard and can improve their learning achievement.

In the current study, the overall students’ perception is improved after implementation of the orientation workshop and engagement. Our results are in agreement with ( Liaw, 2008) **,** ( Krieg and Henson, 2016) **,** ( Carvalho, Areal and Silva, 2011) **,** and ( Green, Inan and Denton, 2012).

( Liaw, 2008) tested the usefulness of Blackboard learning management system and obtain positive results of the Blackboard effectiveness as an online learning tool which improves learners’ satisfaction and behavioral intentions. While ( Krieg and Henson, 2016) reported additional overall positive impact of Blackboard platform satisfaction result regarding the staff beside their students.

( Green, Inan and Denton, 2012) mentioned an important adding factor that was a cause of improvement of students’ satisfaction in their study as they stated that the availability of technical assistance is strongly correlated with student satisfaction. Moreover, ( Carvalho, Areal and Silva, 2011) compared Blackboard platform with other available learning management system and described the superiority of Blackboard.

A study that had been done in King Abdulaziz Univesity by ( Al-Malki, AbdulKarim and Shoie Alallah, 2015) measured the perception and satisfaction of students and faculty about the use of Blackboard as a learning management system. The participant staff in their study gave a positive view regarding two points; Blackboard uses as a platform for learning material submission and delivery to students and its use as a good communication tool. Blackboard was described by them as a flexible tool for submission, arrangement, and modification of the course content as well as a good provider up to date status of learning resources. The limited usefulness of the staff from its use required mandatory tasks of Blackboard awareness program for both students and faculty as they recommended in their final conclusion.

An important task in the current project is the orientation program and pre-implementation workshops for use of Blackboard and its utilities that allow full usefulness and enjoyment of the users. This task is mandatory as a result of many obstacles and challenges that necessitate designing orientation programs with helpful workshops to find solutions that can overcome the underutilization of Blackboard as an online learning community tool as required. These challenges were previously presented by ( Uziak *et al.*, 2018) which are summarized as follows; students, as well as instructors, own readiness and willingness, organization factors, technology factors, the time factor, and keeping with the pace.

Students related factors were the thought of lack of a learning atmosphere in Blackboard, fewer opportunities for discussions with other colleagues and faculty, delayed or even no feedback from teachers. Moreover, the lack of immediate clarification of any questions or concerns which may lead to slow down the learning process ( Birch and Volkov, 2005) & ( Liaw, 2008).

The outcomes of these workshops showed in general positive attitudes toward the use of e-learning software like Blackboard. The students and the faculty in our study were open to this available online learning platform. They described it as a useful but still in need of additional training of its use to obtain a maximum benefit. They appreciated its use as a supplementary tool beside the traditional classroom teaching. Really, there was no problem regarding students’ and instructor readiness and willingness in the application of the Blackboard platform as an online learning tool.

The benefits they obtain included increased availability, quick feedback, improved two-way interactions, tracking, and building skills such as organization, time management, and communications, these targeted benefits were recorded previously by ( Christie and Jurado, 2009) and ( El Zawaidy, 2014).

Regarding the organizational challenges, the E-learning unit in our schools provided the tools and full time training for all staff members in groups as well as in individual manner. Also, they provided all technical facilities to assure full successful training.

In the present study, there is significant positive correlation of the overall students’ perception and satisfaction with their grades in clinical biochemistry as well as their total GPA which means a positive correlation with their academic achievement especially in the survey delivered to them in the post-implementation stage.

Our results are in agreement with ( Means *et al.*, 2013), ( Uziak *et al.*, 2018), ( Eryilmaz, 2015), ( Kintu, Zhu and Kagambe, 2017), ( Liu *et al.*, 2016) and ( Marchalot *et al.*, 2018). All these studies proved the effectiveness of Blackboard and other blended teaching methods, as an e-learning management system in the development of academic achievement.

Collaborative learning applications like Blackboard facilitate and encourage students’ engagement and consequently give a better impact on students’ academic achievement as presented in the current study. This could be explained by higher student-faculty interaction, and better engagement in the effective learning practices which become associated with gains in student learning and development. Moreover, the ease delivery of the learning materials, better students’ instructor communication, rapid constructive feedback and the continuous formative assessment are factors that help students in their learning process and the net result is the higher educational achievement with better scores ( Vaughan, 2014).

Moreover, it could be explained, by using online platforms in general increases students’ comprehension and experience. It also enhances their feeling of responsibility for learning, develops their positive attitudes and provides them with trust and satisfaction regarding their learning abilities ( Aljuaid, Alzahrani and Islam, 2014).

As blackboard is one of the blended teaching tools, its effectiveness in learning and teaching is related to the general impacts of blended teaching which are improving the level of academic achievement, verbal communication and motivation to e-learning, persistence of learning impact, research skills, interaction among learners, improving comprehension, and adjusting alternatives conceptions ( Deperlioglu and Kose, 2013), ( Al-Otaibi, 2017) and ( Ryan *et al.*, 2016). All of these factors participate in raising the academic achievement of students.

Recently in 2019, ( Elsawy and Ahmed, 2019) expose the importance of blackboard virtual classrooms that provide online lectures, work on distance training and help to spread education that can make the learner able to learn very effectively and consequently improve the overall academic achievement and score.

## Conclusion

Based on the currently presented second-year medical students’ perception and satisfaction, we could confirm that the Blackboard online platform is a very successful online line learning community tool. Blackboard platform together with face to face classroom-based learning methods helps students for better learning achievement. Blackboard use helps students for good communication with each other as well as with their faculty, continuous assessment and mentoring of their progress and achievements. Continuous orientation courses could be of importance for both students and faculty to learn them how to get the maximum benefits of its tools. Finally, the online learning community is considered a complementary tool to that of classroom-based learning as it facilitates students/students and students’/faculty communication and minimizes the time required for physical contacts.

## Take Home Messages

• Utilizing the online learning management system as “Blackboard”, should be considerd as a tool for supporting teaching and learning process in medical education.
• Continuous training courses and orientation workshops are essential as a part of a professional faculty development program to be capable of activating online learning and teaching various courses.
• Providing equipped classrooms with the technologies is essential for the online learning management system (Blackboard).

## Notes On Contributors

**Ayman Z. Elsamanoudy:** Professor of Medical Biochemistry and Molecular Biology, Faculty of Medicine, Mansoura University, Mansoura, Egypt and Clinical Biochemistry, Faculty of Medicine, King Abdulaziz University, Jeddah, Saudi Arabia. ORCiD: https://orcid.org/0000-0002-8731-6184

**Fayza F. Al Fayez:** Asistant professor of Clinical Biochemistry, Faculty of Medicine, King Abdulaziz University, Jeddah, Saudi Arabia and head of E Learning unit, college of Medicine, King Abdulaiz University.

**Aliaa Alamoudi:** Asistant professor and supervisor of Clinical Biochemistry department, female section, Faculty of Medicine, King Abdulaziz University-Jeddah-Saudi Arabia. ORCiD: https://orcid.org/0000-0001-5428-8853

**Mohammed Hassanien:** Consultant of vice Presidence for educational Affairs and head of assessment unit College of Pharmacy, King Abdulaziz University, Jeddah, Saudi Arabia and Assocaite professor of Medical Biochemistry, College of Medicine, Tanta University, Tanta, Egypt. ORCiD: https://orcid.org/0000-0001-6559-9710
