# Supplementary material for: Enhancing students’ engagement on using Blackboard as an online learning community tool in Clinical Biochemistry
Source: MedEdPublish (2016). 2020 Apr 20;9:71. [Version 1] doi: 10.15694/mep.2020.000071.1 (PMC10697440; doi:10.15694/mep.2020.000071.1)
Supplement: Peer review history

# Reviewer report: Reviewer response for version 1

**DOI:** 10.21956/mep.19993.r30433

**Published:** 2021-01-04

**Authors:** Barbara Jennings

**Affiliations:** 1 Norwich Medical School

This review has been migrated. The reviewer awarded 4 stars out of 5 Thank you to the authors for this interesting research article that investigates the use of workshops for faculty and learner development in e-learning and teaching. The impetus for developing effective digital and online medical education has never been greater. The authors of this paper explore the functionality of, and satisfaction with, a widely-used learning management system; Blackboard.I appreciated the focus within the project on the whole learning community; i.e. faculty awareness and engagement as well as learner outcomes and satisfaction. Though survey data was collected and thoroughly analysed from second year medical students studying clinical biochemistry, the intervention steps included workshops for both faculty and students.The project objectives and methodology are clearly described in the methods section; including research governance, data collation and statistical analyses. A lot of comparisons were made and parameters considered – did the authors make any statistical adjustments for multiple comparisons?The data tables in the results section are clearly and succinctly presented.In their discussion, the authors assert that ‘an LMS is to supplement traditional lecture course content’ – I think this is an outdated perception (and a 2006 citation is used to support it). We need to be much more ambitious for an interactive LMS IMO, particularly in the current climate of medical education with both the pandemic and a global expansion of clinical training.However, this is a useful article and I found the impact of the interventions on satisfaction; awareness, and learning-gain very encouraging.

Reviewer Expertise:

NA

No decision status is available

# Reviewer report: Reviewer response for version 1

**DOI:** 10.21956/mep.19993.r30438

**Published:** 2020-10-15

**Authors:** Abir El Sadik

This review has been migrated. The reviewer awarded 4 stars out of 5 A very interesting and valuable article. It deals with a very important subject which is the learning management system; the Blackboard. In this era of COVID-19 and the lockdown of face-to-face teaching, the Blackboard became a very necessary tool for education. The study highlighted the benefits and challenges encountered by the staff and students, focusing on the biochemistry discipline.

Reviewer Expertise:

NA

No decision status is available

# Reviewer report: Reviewer response for version 1

**DOI:** 10.21956/mep.19993.r30435

**Published:** 2020-06-26

**Authors:** Ken Masters

**Affiliations:** 1 Sultan Qaboos University

This review has been migrated. The reviewer awarded 3 stars out of 5 An interesting paper on enhancing students’ engagement on using Blackboard as an online learning community tool in Clinical Biochemistry. This is especially important as more courses move to an online environment.There are, however, several issues that need to be addressed:• It is not a good idea to supply a link to the questionnaire form for inspection, as links can be broken, and many readers are loath to click on a link that might be malicious. It would be better if the authors could supply a copy of the questionnaire as an appendix or a supplementary file. • Across the paper, there appears to be a confluence of the tool and the community, which is not appropriate. There are even statements speaking of “good awareness of the use of Blackboard as an online learning community in…” The paper needs to clearly distinguish between Blackboard as a tool, and an online learning community that uses Blackboard (and other tools) for teaching and learning.• This problem of confluence has translated into another problem in the way that the study has been conducted, and, perhaps, the way the course has been run. A strong PLC relies heavily on collaboration and sharing – that is one of the things that distinguishes a learning community from an audience that merely receives data from the teacher and completes quizzes. In this study, however, it appears that only one collaborative tool (the forum) was used, and there is only one question on it in the survey. (It is good to see the high use of it, but it is one tool only). There is no mention of other collaborative tools (e.g. wikis, external social media, etc.) that would really need to be used if the class were to be considered a community. As one cannot change the study and the results at this stage, I recommend that the authors acknowledge this as a limitation, and undertake to increase the use of collaborative tools in order to develop the community in future renditions of the course. • There is also some repetition in the paper (e.g. that Blackboard is one of the most commonly-used LMSs).• There are many small language errors in the paper, and sometimes they interfere with the meaning. For instance, the meaning of this sentence is not clear (would certainly require a supporting citation): “The use of e-learning has been dominated by many educational organizations.” Many other sentences are also difficult to understand, and I would strongly recommend that the authors have the paper proof-read very carefully and corrected, before submitting Version 2.So, a reasonably good study, but the paper does need some re-working. I look forward to Version 2 in which these issues are addressed.

Reviewer Expertise:

NA

No decision status is available

# Reviewer report: Reviewer response for version 1

**DOI:** 10.21956/mep.19993.r30437

**Published:** 2020-06-02

**Authors:** Gehan Sadek

**Affiliations:** 1 Menoufia Faculty of Medicine

This review has been migrated. The reviewer awarded 5 stars out of 5 This is a well-structured and informative work. I was satisfied by students’ perception in the post-implementation phase. This work highlights the importance of capacity building of both academic staff and students to be able to start inevitable online teaching. Besides, it attracts attention to the fact of blended learning as a method of learning in the post-COVID 19 period. Very good work.

Reviewer Expertise:

NA

No decision status is available

# Reviewer report: Reviewer response for version 1

**DOI:** 10.21956/mep.19993.r30436

**Published:** 2020-05-29

**Authors:** Zienab Alrefaie

**Affiliations:** 1 King Abdulaziz University

This review has been migrated. The reviewer awarded 5 stars out of 5 This research highlighted the importance of technology blended learning, and the necessity of both faculty and student orientation to embrace technology use in learning . It seems like authors were a step a head concerning the COVID-19 shift into totally technology dependent learning. Their results showed enhanced students’s satisfaction and achievement on further utilizing the learning management system, which help us feel optimistic towards the current situation.

Reviewer Expertise:

NA

No decision status is available

# Reviewer report: Reviewer response for version 1

**DOI:** 10.21956/mep.19993.r30434

**Published:** 2020-04-29

**Authors:** James Gray

**Affiliations:** 1 University of Sheffield

This review has been migrated. The reviewer awarded 4 stars out of 5 In the current Covid19 situation this is an interesting piece of work as many will be falling back to increasing reliance on the supporting digital platforms of their HEIs. I think a key take home is around getting faculty support and development in place as whilst students may be engaging it requires the effective staff support and use to really drive the effectiveness of the platform as a whole

Reviewer Expertise:

NA

No decision status is available

# Reviewer report: Reviewer response for version 1

**DOI:** 10.21956/mep.19993.r30431

**Published:** 2020-04-29

**Authors:** Samar Ahmed

**Affiliations:** 1 Ain Shams University Faculty of Medicine

This review has been migrated. The reviewer awarded 5 stars out of 5 I think this is exceptional work done by an exceptional team. I am happy to see this amount of engagement and intervention. Your work is replicable and carries a message and toolbox for others guided by structured results. Congratulations

Reviewer Expertise:

NA

No decision status is available

# Reviewer report: Reviewer response for version 1

**DOI:** 10.21956/mep.19993.r30432

**Published:** 2020-04-21

**Authors:** Puja Dulloo

**Affiliations:** 1 Pramukhswami Medical College

This review has been migrated. The reviewer awarded 4 stars out of 5 I enjoyed reading the article. This article is need of an hour were face to face connectivity for teaching can be reduced to a great extent. Online Learning Management System (LMS) is required in present set-up. Conducting an orientation workshop by the authors for the new online tool for faculty and students was really good. Inability of using the portal to its maximum would lead to decrease satisfaction rate and interest to work on the platform on either end.Results showed significant correlation for grades in clinical biochemistry course although the percentage of total GPA >4.5 was less (42.5%) than the pre-implementation (52.1%) but total GPA between 3.5-4.49 percentage value increased in post-implementation (53.5%) compared to pre-implementation score (43.3%).Traditional approach may not always have great interaction among the learners or with their faculty member. This aspect of contacting faculty at their flexibly time frame is possible with E-learning tool. When learning becomes enjoyable then students have a deeper understanding of the concept.What I take from this article is that every health institute should have online LMS along with the traditional teaching approach, allowing students to discuss and interact better with peer as well as faculty member.

Reviewer Expertise:

NA

No decision status is available
